# Comparison of Therapies in MS Patients After the First Demyelinating Event in Real Clinical Practice in the Czech Republic: Data From the National Registry ReMuS

**DOI:** 10.3389/fneur.2020.593527

**Published:** 2021-01-12

**Authors:** Zbyšek Pavelek, Lukáš Sobíšek, Jana Šarláková, Pavel Potužník, Marek Peterka, Ivana Štětkárová, Pavel Štourač, Jan Mareš, Pavel Hradílek, Radek Ampapa, Markéta Grünermelová, Marta Vachová, Eva Recmanová, Francesco Angelucci, Simona Halúsková, Martin Vališ

**Affiliations:** ^1^Department of Neurology, Faculty of Medicine and University Hospital Hradec Králové, Charles University in Prague, Hradec Králové, Czechia; ^2^Department of Neurology, Faculty of Medicine and University Hospital Plzen, Charles University in Prague, Plzeň, Czechia; ^3^Third Faculty of Medicine, Charles University and Hospital Kralovské Vinohrady, Charles University in Prague, Prague, Czechia; ^4^Department of Neurology, University Hospital and Masaryk University, Brno, Czechia; ^5^Department of Neurology, Faculty of Medicine, Palacky University and University Hospital Olomouc, Olomouc, Czechia; ^6^Clinic of Neurology, University Hospital Ostrava, Ostrava, Czechia; ^7^Department of Neurology, Hospital of Jihlava, Jihlava, Czechia; ^8^Department of Neurology, Thomayer Hospital, Prague, Czechia; ^9^Department of Neurology, KZ a.s., Hospital Teplice, Teplice, Czechia; ^10^Department of Neurology, Tomas Bata Regional Hospital, Zlín, Czechia; ^11^Memory Clinic, Department of Neurology, Second Faculty of Medicine, Charles University and Motol University Hospital, Prague, Czechia

**Keywords:** multiple sclerosis, treatment, long-term therapy, clinical practice, DMD

## Abstract

**Background:** Multiple sclerosis (MS) is a chronic inflammatory and neurodegenerative disease of the central nervous system. Well-established drugs used for MS patients after the first demyelinating event in the Czech Republic include glatiramer acetate (GA), interferon beta-1a (IFNβ-1a), IFN beta-1b (IFNβ-1b), peginterferon beta-1a (peg-IFNβ-1a), and teriflunomide.

**Objective:** The objective of this observational study was to compare the effectiveness of the abovementioned drugs in patients with MS who initiated their therapy after the first demyelinating event. Patients were followed for up to 2 years in real clinical practice in the Czech Republic.

**Methods:** A total of 1,654 MS patients treated after the first demyelinating event and followed up for 2 years were enrolled. Evaluation parameters (endpoints) included the annualized relapse rate (ARR), time to next relapse, change in the Expanded Disability Status Scale (EDSS) score, and time of confirmed disease progression (CDP). When patients ended the therapy before the observational period, the reason for ending the therapy among different treatments was compared.

**Results:** No significant difference was found among the groups of patients treated with IFNβ-1a/1b, GA, or teriflunomide for the following parameters: time to the first relapse, change in the EDSS score, and the proportion of patients with CDP. Compared to IFNβ-1a (44 mcg), a significant increase in the percentage of relapse-free patients was found for GA, but this treatment effect was not confirmed by the validation analysis. Compared to the other drugs, there was a significant difference in the reasons for terminating GA therapy.

**Conclusion:** Small differences were found among GA, IFNβ and teriflunomide therapies, with no significant impact on the final outcome after 2 years. Therefore, in clinical practice, we recommend choosing the drug based on individual potential risk from long-term therapy and on patient preferences and clinical characteristics.

## Introduction

Multiple sclerosis (MS) is a chronic inflammatory demyelinating disease affecting the central nervous system. The results of clinical studies indicate that timely MS diagnosis and treatment in the initial phase of the disease can significantly hamper its progression, maintain long-term functionality and prevent permanent damage to nervous structures (1). In fact, neuronal loss can occur already in early phases of the disease (2), and if the treatment start is delayed, it results in an irreversible loss of function (3).

Undoubtedly, early initiation of therapy has a crucial impact on MS prognosis. Therefore, the treatment should start immediately after the first demyelinating event to achieve long-term disease remission phases.

Currently, available treatment options include interferon beta (IFNβ)-1a (30 mcg as an intramuscular injection once a week or 44 mcg subcutaneously 3 times a week), glatiramer acetate (GA) (20 mg as a subcutaneous injection daily or 40 mg 3 times a week), IFNβ-1b (250 mcg (1 ml) subcutaneously every other day), peginterferon beta-1a (peg-IFNβ-1a) (125 mcg as a subcutaneous injection once every 2 weeks) and teriflunomide (oral dosing of 14 mg daily).

All these drugs have been shown to have beneficial effects on MS symptoms when administered at early stages. In the REFLEX (REbif FLEXible dosing in early MS) (4) and CHAMPS (Controlled High-Risk Subjects Avonex MS Prevention Study) (5) studies, compared to placebo, IFNβ-1a proved to be effective in reducing the rate of relapse and conversion to clinically definite MS (CDMS). Similar effects were observed with INFβ-1b in the BENEFIT (Betaseron in Newly Emerging MS for Initial Treatment) study (6), while GA (7) and Teriflunomide (8) postponed the second relapse rate in patients with clinical isolated syndrome (CIS). Peginterferon also reduced the adjusted annualized relapse rate in a 2-year follow-up period (9).

Altogether, these studies suggest that these drugs are effective in reducing the risk or the rate of relapse in timely treated patients. However, an in-depth comparison of their therapeutic effects is still not sufficient. Thus, in this study, we enrolled 1.654 MS patients were treated with different drugs after the first demyelinating event and followed up for 2 years. During this time, we evaluated the differences in clinical parameters among these drugs to compare their efficacy and safety. Evaluation parameters included the annualized relapse rate (ARR), time to next relapse, change in the Expanded Disability Status Scale (EDSS) score, and time of confirmed disease progression (CDP). When patients ended the therapy before the observational period, the reason for ending the therapy among different treatments was also compared.

## Materials and Methods

### Study Population

Males and females from 15 to 65 years of age (inclusive) at the beginning of DMD therapy with a diagnosis of CIS or definite CDMS were enrolled in the study. All patients with CIS or definite CDMS enrolled had experienced the first MS attack according to the 2010 McDonald criteria (10, 11). A total of 1,654 patients who satisfied the following inclusion criteria were identified:

- Treatment with IFNβ-1a 30 mcg once a week (384 patients), GA 20 mg daily or 40 mg 3 times a week (509 pts), IFNβ-1b 250 mcg every other day (199 pts), IFNβ-1a 44 mcg 3 times a week (467 pts), peg-IFNβ-1a 125 mcg 0.5 times a week (7 pts), or teriflunomide 14 mg daily (88 pts) initiated after the first relapse.
- The therapy was started within 2 years from the start of the disease.
- Follow-up duration of at least 24 months.

**Figure 1:**
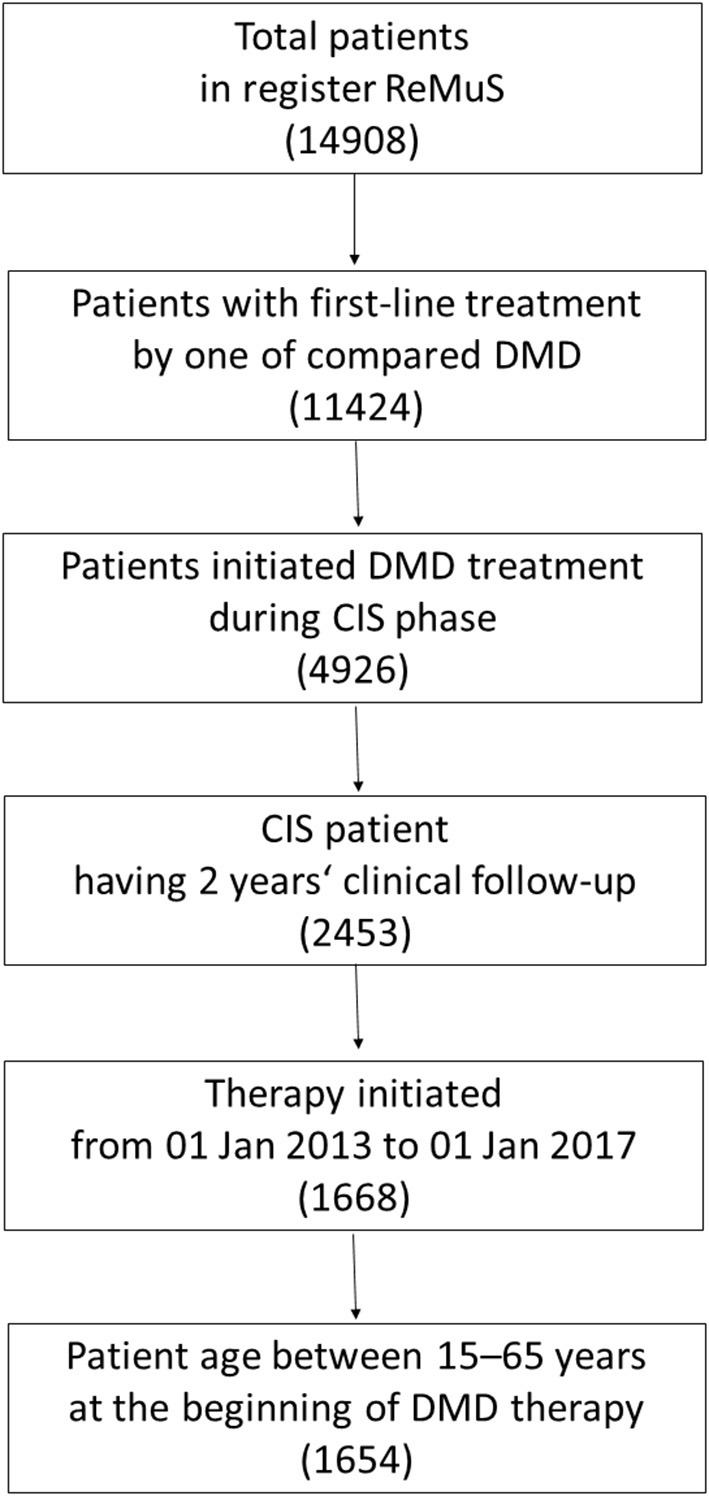
Patient enrolment and inclusion in the study.

Figure 1 (flow chart) summarizes patient enrolment and inclusion in the study. Patients were assigned to one of six treatment groups according to the initial DMD treatment, i.e., no treatment sequencing was allowed. Table 1 shows the characteristics of the treatment groups.

**Table 1:** Demographic and clinical characteristics of the treatment groups.

**Parameter**	**All patients (*n* = 1,654)**	**IFNβ-1a 44 mcg (*n* = 467)**	**GA (*n* = 509)**	**IFNβ-1b (*n* = 199)**	**IFNβ-1a 30 mcg (*n* = 384)**	**Teriflunomide (*n* = 88)**	**Peg-IFNβ-1a (*n* = 9)**	**Statistical simultaneous comparison (except for peg-IFNβ-1a)**		
								**Test**	***P*-value (corr.)**	**Effect size**
Age	34.42 (9.79)	32.832 (9.43)	33.7981 (9.85)	36.7914 (9.21)	34.474 (10.05)	41.12 (7.88)	31.029 (7.07)	*	<0.001	0.04
Disease duration (in months)	5.323 (4.12)	4.665 (3.55)	5.5346 (4.39)	5.9271 (4.01)	5.196 (4.02)	6.49 (4.96)	9.059 (7.58)	**	<0.001	0.02
DMD treatment duration (in months)	18.508 (7.12)	18.528 (7.28)	17.0362 (7.41)	18.3597 (7.29)	20.134 (6.21)	20.51 (6.13)	14.094 (4.99)	***	<0.001	0.03
EDSS score at baseline (0 M)	1.853 (0.89)	2.11 (0.98)	1.78 (0.83)	2.0151 (0.91)	1.529 (0.71)	1.97 (0.88)	1.857 (0.8)	***	<0.001	0.06
EDSS score in the first year (12 M)	1.886 (0.99)	2.164 (1.08)	1.7546 (0.91)	2.018 (0.93)	1.604 (0.89)	2.08 (0.99)	2 (1.15)	***	<0.001	0.05
EDSS score in the second year (24 M)	1.98 (1.08)	2.231 (1.18)	1.8546 (1.02)	2.1332 (1.08)	1.716 (0.96)	2.17 (1.01)	2 (1.15)	***	<0.001	0.04
Absolute change in EDSS score after 1 year	0.034 (0.61)	0.047 (0.62)	−0.0092 (0.59)	0.0052 (0.57)	0.071 (0.66)	0.11 (0.43)	0.143 (0.38)	*	0.295	<0.01
Absolute change in EDSS score after 2 years	0.128 (0.72)	0.121 (0.74)	0.0747 (0.7)	0.1181 (0.78)	0.188 (0.74)	0.24 (0.6)	0.143 (0.38)	*	0.924	<0.01
Time to CDP (in months)	10.642 (4.84)	11.96 (4.92)	9.859 (5.55)	10.2418 (3.39)	10.331 (4.55)	8.44 (4.4)	NA	**	0.675	0.03
Number of relapses	0.584 (0.96)	0.707 (1.01)	0.5069 (0.94)	0.5327 (0.89)	0.591 (1)	0.5 (0.87)	0.143 (0.38)	***	0.009	0.01
ARR	0.282 (0.46)	0.341 (0.48)	0.2453 (0.46)	0.2579 (0.44)	0.284 (0.47)	0.24 (0.42)	0.069 (0.18)	***	0.008	0.01
Time to next relapse (in months)	10.291 (7.12)	9.895 (6.92)	10.2243 (7.42)	11.3047 (7.3)	10.695 (7.18)	9.03 (6.22)	9.738 (NA)	***	0.663	0.01
Follow-up duration (in months)	24.671 (1.54)	24.684 (1.49)	24.6947 (1.56)	24.7055 (1.53)	24.63 (1.54)	24.66 (1.73)	23.415 (1.86)	***	0.939	<0.01
**Gender**								****	0.002	0.131
Females	1157 (69.95)	312 (66.81)	399 (78.39)	125 (62.81)	265 (69.01)	53 (60.23)	3 (42.86)			
Males	497 (30.05)	155 (33.19)	110 (21.61)	74 (37.19)	119 (30.99)	35 (39.77)	4 (57.14)			
**CDP**								****	0.406	0.054
No	1566 (94.68)	437 (93.58)	489 (96.07)	191 (95.98)	360 (93.75)	82 (93.18)	7 (100)			
Yes	88 (5.32)	30 (6.42)	20 (3.93)	8 (4.02)	24 (6.25)	6 (6.82)	0 (0)			
**Relapse status during follow-up**								****	0.034	0.089
Relapse occurred	603 (36.46)	198 (42.4)	165 (32.42)	68 (34.17)	142 (36.98)	29 (32.95)	1 (14.29)			
Relapse free	1051 (63.54)	269 (57.6)	344 (67.58)	131 (65.83)	242 (63.02)	59 (67.05)	6 (85.71)			
**Reason for treatment discontinuation**								****	0.002	0.13
Adverse Event	80 (9.38)	33 (12.64)	13 (4.64)	9 (8.49)	23 (12.37)	2 (10.53)	0			
Lack of efficacy	350 (41.03)	122 (46.74)	78 (27.86)	55 (51.89)	82 (44.09)	13 (68.42)	0			
Lack of tolerance	313 (36.69)	81 (31.03)	153 (54.64)	25 (23.58)	52 (27.96)	2 (10.53)	0			
Pregnancy	110 (12.9)	25 (9.58)	36 (12.86)	17 (16.04)	29 (15.59)	2 (10.53)	1 (100)			
no info	801	206	229	93	198	69	6			

Numerical variables are expressed as the means (standard deviations), and categorical variables (gender, CDP, relapse-free status, reasons for treatment discontinuation) are expressed as frequency (percentage). The DMD groups were compared using parametric (*, ** after logarithmic transformation) or nonparametric ANOVA (***). Categorical variables were tested using the chi-squared test of independence. Reported P-values [(P value (corr.)] are corrected for multiple testing by Benjamini-Hochberg procedure.

### Study Design

This was a retrospective, controlled, observational, cohort study conducted at 15 centers in the Czech Republic specializing in the diagnosis and therapy of demyelinating diseases. Patients with CIS or definite CDMS initiated therapy in the period from 01 Jan 2013 to 01 Jan 2017. The beginning of 2013 was chosen to include good-quality and prospectively controlled data.

The data were collected from the Czech nationwide registry of MS patients (ReMuS). As of 31 December 2018, ReMuS included information on 14,908 patients with at least 1 visit to an MS center from 2010. ReMuS was established by the Endowment Fund IMPULS (www.multiplesclerosis.cz) in cooperation with the Liquorology and Neuroimmunology Section of the Czech Neurological Society, Czech Medical Association of J. E. Purkyně as the specialized guarantor of the project. The purpose of the registry is to provide good-quality data on the epidemiology, diagnosis and severity of MS, as well as on therapy and adverse effects.

Data in registry ReMuS have been prospectively controlled since 2013, which ensures good data quality. Clinical data of patients on DMD treatment are regularly entered in the registry for each visit in the MS center (at least one data entry for each patient every half year).

### Clinical Parameters (Endpoints)

The following clinical parameters were evaluated:

1. Annualized absolute EDSS score change (dEDSS)
2. Rates and time to the development of CDP
3. Annualized relapse rate (ARR)
4. Time to the next (first following DMD therapy administration) relapse
5. Reasons for terminating the therapy.

EDSS scores prior to therapy were required to be stable for at least 3 months and were acquired at the beginning of treatment and every 6 months.

Relapses were defined according to the following diagnostic and therapeutic guidelines: (1) newly developing symptoms or reactivation of pre-existing neurological deficits for a minimum of 24 h in the absence of increased body temperature or infections and (2) symptoms occurring at least 30 days after the preceding episode.

The CDP was defined according to the changes in the EDSS score sustained over 6 months (1.0-point increase or greater if the EDSS score was more than 0.0 at baseline, or 1.5-point increase or greater if the EDSS score was 0.0 at baseline).

Reported reasons for terminating the therapy included therapy intolerance (defined as the presence of intolerant side effects, such as heart problems, liver problems, signs of infection or allergic reactions), lack of effectiveness, occurrence of adverse events, and pregnancy.

### Standard Protocol Approvals, Registrations, and Patient Consent

The ReMuS register works on the basis of informed consent, an integral appendix that specifies the scope of the study and determines the possible area of data collection. This informed consent form is approved by all 15 ethical committees for all 15 MS centers in the Czech Republic. Based on this informed consent, it is possible to use retrospective data for scientific and research purposes without requiring new approvals. All patients signed an informed consent form for inclusion in ReMuS.

### Statistical Analysis

Differences in numerical clinical scores among DMD groups were compared using parametric or non-parametric ANOVA followed by a *post-hoc* paired comparison, which was performed using parametric or non-parametric *t*-tests for numerical parameters (descriptive characteristics and endpoints). The choice of a parametric or non-parametric test depended on the distribution of the given scores. For each score, normality was assessed using statistical tests and graphic data analysis. The reason for treatment discontinuity was simultaneously tested by the chi-squared test of independence and other categorical parameters (CDP and relapse status during follow-up) by a logistic regression model. Moreover, the effect size (or Cramer's V for the chi-squared test) for simultaneous testing was reported. Survival functions for the time to relapse and CDP were estimated using the Kaplan-Meier method. Pairwise comparison of the treatment effect (hazard functions) for CDP and relapse occurrence was performed by estimating Cox proportional hazards models and testing hazard ratios with the Wald test criterion.

Patients administered peg-IFNβ-1a were excluded from the statistical comparisons because of the insufficient number of patients (*n* = 7). Thus, this group is reported for descriptive purposes only.

Validation analysis was performed for all endpoints to reduce the risk of selection and confounding biases by employing multiple regression models adjusted for age and EDSS at baseline. The appropriate regression model (OLS, logistic, zero-inflated Poisson model, or Cox proportional hazards model) was chosen based on the distribution of endpoints. The Cox model enables simultaneous evaluation of the effect of several variables (treatment group and confounding factors) on survival. Pairwise comparison of treatment effects in survival analysis was also performed by testing coefficients of Cox models (hazards functions), instead of testing the survival functions using the log-rank test, to perform a consistent method in the validation analysis.

Interaction analysis was performed to confirm the real treatment effect for those endpoints whose results (significance of pairwise comparison) differed between pairwise comparisons without adjustment for confounding factors and the same comparison with adjustment (validation analysis). The interaction analysis was performed as linear regression modeling of the investigated endpoint on the interaction of confounding factors (EDSS and age at baseline) and treatment.

The hypotheses (differences among and between groups) were tested at the *P* ≤ 0.05 two-tailed significance level after controlling the false discovery rate with the Tukey procedure for pairwise comparison and the Benjamini-Hochberg procedure for simultaneous testing and for the validation analysis. We also compared 95% confidence interval estimates and effect sizes. All statistical analyses were performed using the R project for statistical computing version 3.5.3 (www.r-project.org).

## Results

### Descriptive Statistics of the Studied DMD Groups

The mean age (standard deviation) of all patients was 34.4 (9.8) years; the disease duration at baseline (start of the first DMD therapy) was 5.3 (4.1) months; the duration of the first DMD therapy was 18.5 (7.1) months; and the EDSS score was 1.8 (0.9).

The groups differed in their age. Patients treated with teriflunomide were significantly older than the other patients (mean age 41.1 years). The mean age of the remaining 4 groups ranged between 32.8 and 36.8 years. The groups were not balanced in gender (percentage). The GA group included 78% females, a rate considerably higher than 60% in the teriflunomide group and 63% in the IFNβ-1b group. IFNβ-1a 44 mcg (67%) and IFNβ-1a 30 μg (69%) were in between. The patient groups also differed in the mean disease duration at baseline: IFNβ-1a 44 mcg was administered after a mean of 4.7 months from onset of the disease, while teriflunomide was administered after 6.5 months. The other DMDs were prescribed during month 5. Additionally, the duration of the given DMD therapy differed. GA therapy duration was shorter (17 months on average) than that of the other DMDs (18–20 months on average). The mean (median) EDSS score at baseline and at 12 and 24 months also differed among groups. The lowest EDSS score at baseline was found for the IFNβ-1a 30 mcg [1.5 (1.5)] and GA groups [1.8 (1.5)]. In the remaining 3 groups, the mean EDSS score at baseline ranged from 2.0 to 2.1 (median 2.0). After 2 years, the mean EDSS scores increased in all groups: 1.7 (IFNβ-1a 30 mcg), 1.9 (GA), and 2.1–2.2 in the remaining groups. The group characteristics and statistical comparisons are reported in Table 1. The results of the pairwise comparisons are shown in Table 2. From statistical comparison, the Peg-IFNβ-1a group was excluded because of the limited number of patients available (7 patients).

**Table 2:** Pairwise comparison of clinical parameters among treatment groups.

**Parameter**	***Post-hoc*** **paired comparison of the DMD groups [*****P-*****values (corr.)]**									
	**2 vs. 1**	**3 vs. 1**	**4 vs. 1**	**5 vs. 1**	**3 vs. 2**	**4 vs. 2**	**5 vs. 2**	**4 vs. 3**	**5 vs. 3**	**5 vs. 4**
Age	0.1	<0.001	0.04	<0.001	<0.001	0.4	<0.001	0.002	0.00011	<0.001
Disease duration (in months)	0.01	<0.001	0.2	0.003	0.3	0.9	0.2	0.1	0.981	0.1
DMD treatment duration (in months)	0.001	0.5	0.01	0.04	0.04	<0.001	<0.001	0.009	0.03	0.6
EDSS at baseline (0M)	<0.001	0.4	<0.001	0.1	0.001	<0.001	0.2	<0.001	0.3	<0.001
EDSS in the first year (12M)	<0.001	0.3	<0.001	0.5	<0.001	0.01	0.01	<0.001	0.9	<0.001
EDSS in the second year (24M)	<0.001	0.7	<0.001	0.9	<0.001	0.03	0.01	<0.001	0.9	<0.001
Absolute change of EDSS after 1 year^a^	0.6 (0.9)	0.9 (0.4)	0.9 (0.6)	0.8 (0.9)	0.9 (0.8)	0.3 (0.8)	0.4 (0.5)	0.7 (0.9)	0.6 (0.8)	0.9 (0.9)
Absolute change of EDSS after 2 years^a^	0.9 (0.5)	0.9 (0.7)	0.9 (0.9)	0.9 (0.9)	0.9 (0.9)	0.9 (0.5)	0.9 (0.5)	0.9 (0.9)	0.9 (0.9)	0.9 (0.9)
Time to CDP (in months)^a^	0.6 (0.8)	0.9 (<0.001)	0.9 (0.8)	0.7 (0.8)	0.9 (0.8)	0.9 (0.8)	0.9 (0.8)	0.9 (0.8)	0.9 (0.8)	0.9 (0.8)
Number of relapses^c^	0.003 (0.9)	0.1 (0.9)	0.1 (0.9)	0.1 (0.9)	0.7 (0.9)	0.2 (0.9)	0.9 (0.9)	0.6 (0.9)	0.8 (0.9)	0.6 (0.9)
ARR^c^	0.002 (0.9)	0.1 (0.9)	0.1 (0.9)	0.1 (0.9)	0.8 (0.9)	0.1 (0.9)	0.8 (0.9)	0.6 (0.9)	0.8 (0.9)	0.6 (0.9)
Time to next relapse (in months)^a^	0.8 (0.8)	0.6 (0.6)	0.6 (0.6)	0.7 (0.6)	0.6 (0.6)	0.7 (0.6)	0.7 (0.6)	0.7 (0.7)	0.6 (0.6)	0.6 (0.6)
Follow-up duration in (months)	0.9	0.9	0.9	0.9	1	0.9	0.9	0.9	0.9	0.9
CDP^b^	0.2 (0.3)	NA	0.917 (0.7)	NA	NA	0.174 (0.2)	NA	NA	NA	NA
Relapse status during follow-up^b^	0.013 (0.261)	0.238 (0.812)	0.270 (0.812)	0.270 (0.876)	0.819 (0.876)	0.310 (0.135)	0.921 (0.876)	0.719 (0.490)	0.921 (0.812)	0.719 (0.812)
**Reason for treatment discontinuation^b^**										
Adverse Event	0.004 (0.007)	NA	0.5 (0.5)	NA	NA	0.018 (0.030)	NA	NA	NA	NA
Lack of efficacy	<0.001 (0.006)	0.8 (0.4)	0.2 (0.8)	0.050 (0.3)	<0.001 (0.002)	0.050 (0.029)	0.9 (0.9)	0.12 (0.3)	0.050 (0.1)	0.2 (0.3)
Lack of tolerance	<0.001 (<0.001)	0.2 (0.1)	0.2 (0.1)	NA	<0.001 (<0.001)	<0.001 (<0.001)	NA	0.7 (0.9)	NA	NA
Pregnancy	0.5 (0.5)	0.5 (0.2)	0.5 (0.4)	NA	0.8 (0.5)	0.8 (0.7)	NA	0.8 (0.5)	NA	NA
Survival analysis for CDP^d^	0.5 (0.4)	0.5 (0.4)	0.9 (0.9)	0.9 (0.9)	0.9 (0.9)	0.5 (0.4)	0.5 (0.4)	0.5 (0.4)	0.5 (0.4)	0.9 (0.9)
Survival analysis for relapse occurrence^d^	0.010 (0.2)	0.2 (0.7)	0.3 (0.7)	0.3 (0.8)	0.9 (0.9)	0.3 (0.1)	0.9 (0.8)	0.7 (0.3)	0.9 (0.7)	0.8 (0.8)

*Firstly, post-hoc paired comparison of the groups (in two types of parameters: descriptive characteristics and endpoints) was done using t-tests or non-parametric t-tests with Tukey correction for the false discovery rate for numerical parameters. Paired comparison for categorical parameters (CDP, Relapse status during follow-up, Reason for treatment discontinuation) was run by logistic regression model*.

*Pairwise comparison of hazard functions for CDP (time to CDP) and Relapse status (time to first relapse) was performed by estimating Cox proportional hazards models and testing hazard ratio with the Wald test criterio*.

Further, validation analysis of paired comparison of the groups for each endpoint was performed to reduce selection biases by employing multiple regression models adjusted for age and EDSS at baseline. The appropriate regression model (

a *OLS*,

b *logistic*,

c *zero-inflated Poisson model*,

d Cox-proportional hazard model

*) was chosen based on the distribution of the selected endpoint. The presented output from validation analysis are P values in parentheses. P values from validation analysis and pairwise hazard ratio comparison are corrected for multiplicity by Benjamini-Hochberg procedure*.

*Discrepancy between paired comparison without and with adjustment for age and EDSS is highlighted by red color. The pairwise comparison of categorical endpoints was done if at least 10 patients were observed (within treatment group and occurrence of the event), otherwise no testing was performed, and it is denoted as “NA”*.

*The DMD group names are abbreviated as: 1 = IFNβ-1a 44mcg, 2 = GA, 3 = IFNβ-1b, 4 = IFNβ-1a 30mcg, 5 = teriflunomide*.

### Comparison of EDSS Score Change and CDP Development During the 2-Year Follow-Up Period Among the Groups

**Figure 2:**
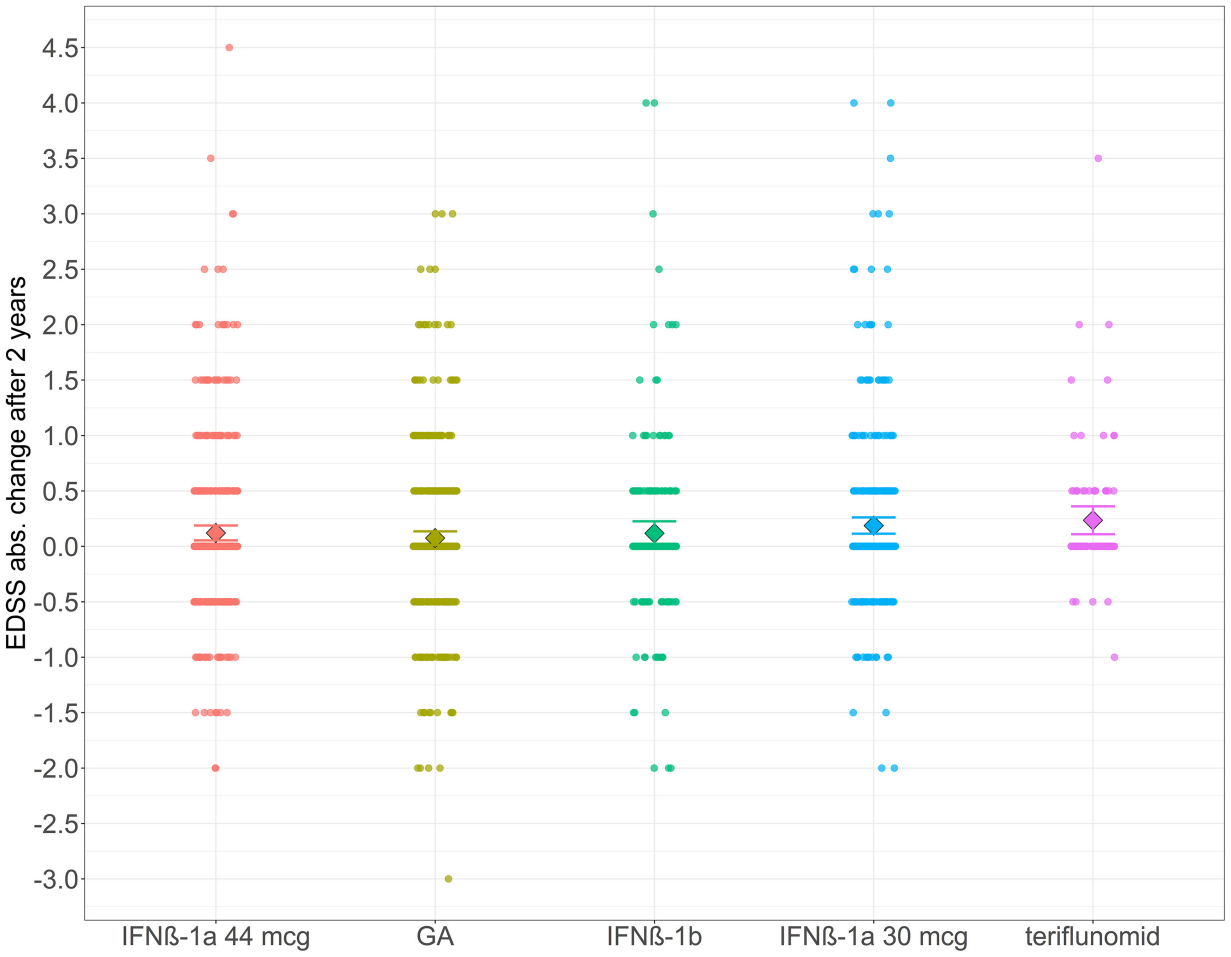
The distribution of absolute change in the EDSS scores 2 years from baseline and the 95% confidence interval estimates of means for the DMD groups. Data points (circles) show all values of 2-year absolute change in EDSS. The rhombus represents the group mean, and error bars depict 95% confidence interval estimates of means for the DMD groups.

Figure 2 shows all values of absolute changes in EDSS score after 2 years and 95% confidence interval estimate of the mean change. The greatest increase in EDSS score was observed for the teriflunomide group, with a mean of 0.24 (median 0). The lowest increase in EDSS score observed for the GA group, with a mean of 0.07 (median 0). The differences between the groups were not significant (*P*-value = 0.869). No group except teriflunomide showed a marked change in EDSS score after the 1st year (mean increase by 0.11).

**Figure 3:**
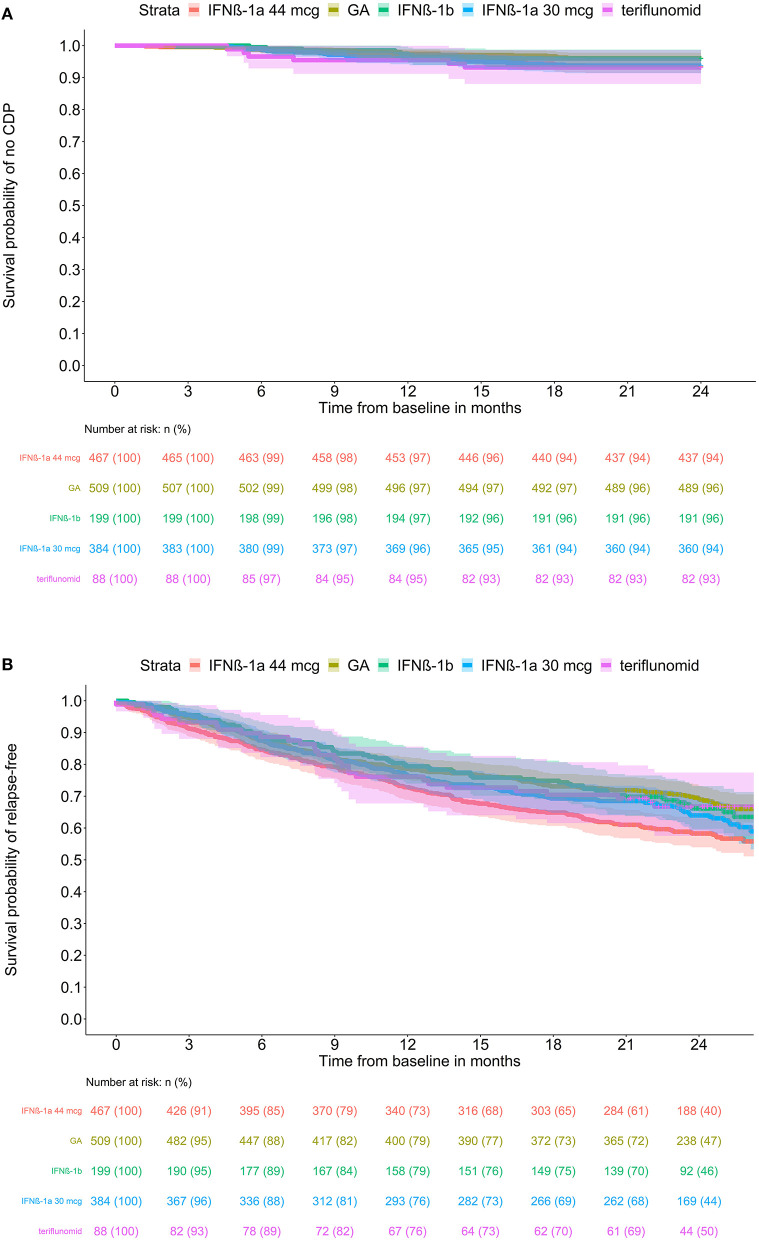
Estimated (K-M) survival functions (with 95% confidence intervals) for **(A)** CSP until month 24 and **(B)** relapse occurrence by treatment groups. Kaplan-Meier estimated survival functions with 95% confidence intervals by treatment group for ARR. The at-risk table contains the number (*n*) and percentage of patients at risk.

CDP (onset and time) and the difference among the groups were analyzed. Unfortunately, the 2-year follow-up period was too short to compare this parameter. CDP occurred only in 88 (5.32%) patients over the 2 years. We thus have insufficient CDP data to formulate an inductive assessment (to generalize the conclusions to a wider population), particularly for teriflunomide (6 times) and IFNβ-1b (8 times). Figure 3 shows the estimated survival function for CDP until month 21 (because of the confirmation period) for each treatment group.

No significant difference was found for CDP percentage among the groups. The lowest CDP percentages were observed for GA (3.9%) and IFNβ-1b (4.0%). A higher percentage was seen for IFNβ-1a 44 mcg (6.4%) and IFNβ-1a 30 mcg (6.3%). Although teriflunomide showed the highest percentage (6.8%), this estimate is based on only 6 cases and is thus not reliable. The pairwise comparison of hazard functions exhibited no significant differences (see corrected and unadjusted *P*-values in Table 2).

### Comparison of Relapse Rates During the 2-Year Follow-Up Period Among Groups

**Figure 4:**
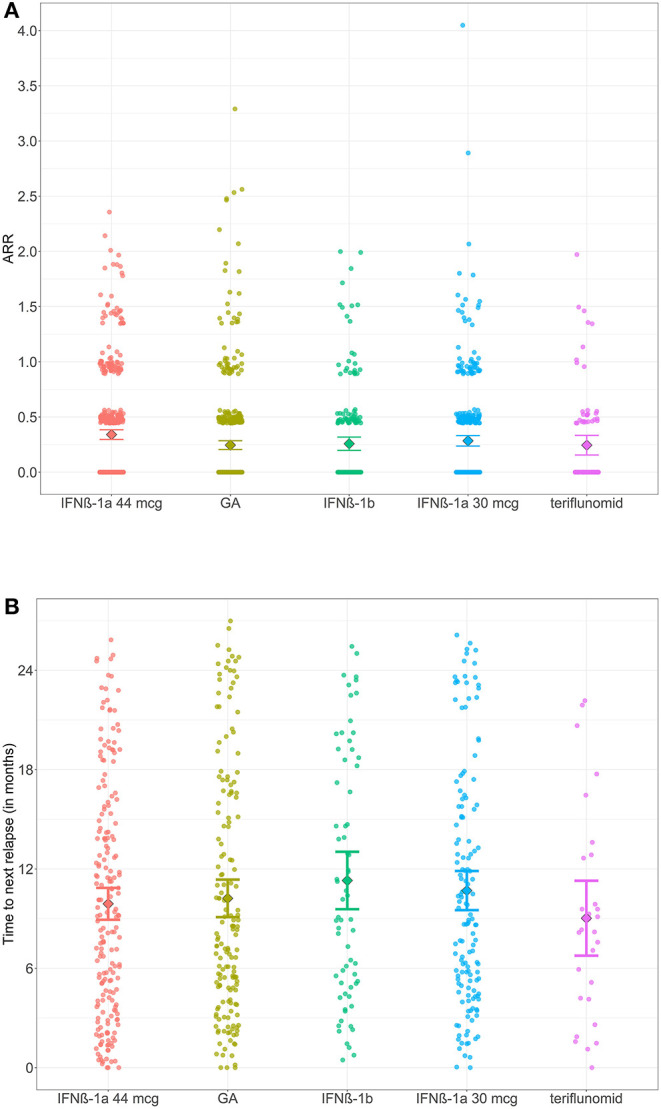
The distribution of times to next relapse and ARR and the 95% confidence interval estimates of means for the DMD groups. Data points (circles) show all values of **(A)** time to next relapse, **(B)** ARR. The rhombus represents the group mean, and error bars depict 95% confidence interval estimates of means for the DMD groups.

The distribution of times to first relapse and ARRs and their 95% confidence interval mean estimates are shown in Figure 4. In all patients, the percentage of patients with at least 1 relapse during the 2-year follow-up period was 36.5%; the mean relapse rate (during the 2-year follow-up) was 0.58 (median 0.00), and the annualized relapse rate (ARR) was 0.28 (0.00). For IFNβ-1a 44 μg, at least 1 relapse occurred in 42.4% of patients; its mean relapse rate of 0.71 (1.0) and ARR of 0.34 (0.0) were the highest and were significantly (*P*-value = 0.002) higher than those of GA [32.4% of patients; mean 0.51 (0.0) and ARR 0.25 (0.0)]. The results for the other groups were similar to those for GA.

The time to the first relapse from baseline (start of DMD therapy) showed no significant differences among the groups (see Figure 4). The shortest time was observed for teriflunomide, with a mean of 9.4 months, and the longest time was observed for IFNβ-1b (11.1 months).

The survival analysis showed significant differences in hazard ratios between IFNβ-1a 44 mcg and GA, estimated hazard function (*P*-value 0.01); see pairwise comparisons in Table 2. Survival functions can be seen in Figure 3B.

### Comparison of the Reasons for Terminating the Therapy

A total of 51.6% of patients terminated the therapy for different reasons. The percentage of patients who stopped therapy was the lowest for teriflunomide (21.6%) and the highest for IFNβ-1a 44 mcg (55.9%). The groups showed a significant difference (*P*-value < 0.001; chi-squared goodness-of-fit-test).

Therapy intolerance, as a result of post-injection complications, was the most common reason in the GA group (54.6%), followed by lack of effectiveness (27.9%), pregnancy (12.9%), and other adverse effects (4.6%).

In the IFNβ-1b group, the most common reason was lack of effectiveness (51.9%), followed by therapy intolerance as a result of post-injection complications (23.6%), pregnancy (16.0%), and other adverse effects (1 case = 8.5%).

In the other 2 groups (IFNβ-1a 44 mcg and 30 mcg), the reasons for terminating the therapy were (relatively) comparable. The most common reasons were lack of effectiveness (44.1–46.7%), followed by therapy intolerance resulting from post-injection complications (28.0–31.0%). The rates of pregnancy and other adverse effects were lower (9.6–15.6%). The logistic regression, used to perform a paired statistical comparison of the different odds ratios of reasons, showed a significant difference in lack of efficacy and tolerance between the GA group and the other groups (IFNβ-1a 44 mcg, IFNβ-1b, and IFNβ-1a 30 mcg). The adverse event as the reason for discontinuation was significantly different between GA and IFNβ-1a 44 mcg and IFNβ-1a 30 mcg.

### Pairwise Comparison After Adjustments for Confounding Factors

Validation analysis was performed for the studied endpoints, and adjusted models for EDSS at baseline and age were used for this purpose. Outputs (*P*-values) from validation analysis are reported in Table 2 in parentheses.

No significant differences were found between any DMD groups for the EDSS score/CDP changes over 2 years in non-adjusted analysis, but validation analysis suggests that there is a significantly greater time to CDP of ~1.7 months between IFNβ-1a 44 mcg (11.96 months on average) and IFNβ-1b (10.24 months) if CDP occurred. However, the occurrence of CDP was more frequent 30x (6%) in the IFNβ-1a 44 mcg group than 8x (4%) in the IFNβ-1b group. Only 8 occurrences (and values for time to CDP) in the IFNβ-1b group were included in the sensitivity analysis to identify whether the treatment effect on the time to CDP may be real. Validation of hazard functions confirms the conclusion that there is no treatment effect on CDP.

For the rate of relapse during the 2-year follow-up period (and its annualized form, i.e., ARR), differences were identified between GA and IFNβ-1a 44 mcg (*P*-value = 0.002; see Figure 4). For GA, a higher percentage of patients was relapse-free (67.6%) than IFNβ-1a 44 mcg (57.6%) for 2 years. Validation of significance was performed using a zero-inflated Poisson regression model for the number of relapses and its annualized derivative (ARR) over 2 years. We found that the difference between the groups ceased to be significant after including age and EDSS score at baseline (*P* = 0.964, 0.999). The survival analysis indicated the same conclusion: there was no difference in the treatment effect between GA and IFNβ-1a 44 mcg (*P*-value 0.218) on the occurrence and time to the first relapse.

The discrepancy in significance led us to explore the interaction of treatment, age and EDSS on the occurrence of relapses represented by the ARR endpoint. A linear regression model with the interaction terms (without one influential, outlier observation with ARR > 3) infers that only age (*P*-value = 0.039) has an effect on ARR. The estimated interaction models (OLS1 for all observations and OLS2 without the outlier) can be seen in Supplementary Table 1 and in Supplementary Figure 1A for all patients (IFNβ-1a 44 mcg and GA), Supplementary Figure 1B for all patients in these two groups except for one patient with an influential (outlier) ARR value equal to 3.3. After excluding this influential observation, there was no significant effect of treatment (*P*-value = 0.2306) on ARR after adjustment for age and EDSS score. In addition, the interaction analysis (Supplementary Figure 1B and Supplementary Table 1) shows a significantly negative relationship between age and ARR (*P*-value 0.0391). Older patients (50+) generally had a lower ARR than younger patients, which confirms the known fact that the relapse activity of the disease decreases in the long term.

Validation analysis confirms the pairwise significant differences in the reasons for discontinuation.

## Discussion

This analysis, taking advantage of real clinical practice data in the Czech Republic, focused on 1,654 patients with clinically isolated MS who started treatment with IFNβ, peg-IFNβ, GA or teriflunomide. Only the groups of patients under IFNβ, GA and teriflunomide treatments were analyzed in detail. The patient group with peg-IFNβ included only 7 patients and thus could not be used to formulate significant conclusions.

The results showed that the patient groups treated with IFNβ, GA or teriflunomide showed no significant differences in the clinical parameters investigated, i.e., the time to first relapse, EDSS score change and the proportions of patients with CDP. Nonetheless, small variations in some of these parameters were noted. The limitation of this study is the lack of paraclinical data, especially MRI data, but these data have not been available.

The time to first relapse after starting DMD therapy was shortest for teriflunomide (with a mean of 9.4 months) and longest for IFNβ-1b (with a mean of 11.1 months).

Pairwise comparison (without adjustment for confounding factors) indicates the treatment effect between IFNβ-1a 44 mcg and GA in the occurrence of relapses in the first 2 years. The occurrence of relapse was 42.4%, and the ARR was 0.34 in the IFNβ-1a 44 mcg group. In the GA group, the occurrence of relapse was lower (32.42%) and ARR = 0.25. The IFNβ-1a 44 mcg group had a 0.5 higher EDSS score at baseline (2.1 on average, median equals 2.0) than the GA group (1.8, 1.5). Both groups were comparable by age (32.8 vs. 33.8 on average). The validation analysis showed that compared to IFNβ-1a 44 mcg (57.6%), a higher proportion of relapse-free patients (67.6%) in the group of patients on GA for 2 years was influenced by age, but there was no significant difference in the treatment effect.

The greatest increase in EDSS score was seen in the groups on teriflunomide and GA. The lowest CDP rates were observed for GA (3.9%) and IFNβ-1b (4.0%). Higher rates were seen in IFNβ-1a 44 mcg (6.4%) and IFNβ-1a 30 mcg (6.3%). No significant differences were found between any DMD groups for the EDSS score/CDP changes over 2 years in non-adjusted analysis, but validation analysis suggests that there is a significantly higher time to CDP of ~1.7 months between IFNβ-1a 44 mcg (11.96 months on average) and IFNβ-1b (10.24 months) if CDP occurred (30x in IFNβ-1a 44 mcg, 8x IFNβ-1b). Compared to the subgroup of 30 patients with CDP from IFNβ-1a 44 mcg with average age 35.7 and EDSS (2.5, 3.0), the subgroup of 8 patients with CDP from the treatment group IFNβ-1b are ~4.6 months older (average age equals to 40.3) and in better clinical conditions with EDSS (2.1 in average, median = 2.5). Based on this comparison, it seems that CIS patients with IFNβ-1b as first-line treatment have a lower risk of CDP (4.0%) but with a shorter time to occurrence (10.24 months) during the 2-year follow-up, followed by patients treated with IFNβ-1a 44 mcg (6%, 11.96 months). CDP occurs in older patients (~40.3 years) with lower EDSS (with a centroid between 2 and 2.5) administered IFNβ-1b compared to younger (35.7 years) patients with higher EDSS (with a centroid of 2.5–3.0) treated with IFNβ-1a 44 mcg. More patients are required to confirm statistically this interaction of treatment effect, age and EDSS to CDP.

Our study suggests that there are no relevant differences among the effects of these drugs on clinical parameters. These data are partially in line with similar studies. Available head-to-head studies analyzed the treatment of patients with relapsing-remitting MS (RRMS), but their results were not consistent. The EVIDENCE study showed a significantly lower relapse rate in patients on IFNβ-1a 44 mcg and, at the same time, a reduced relapse rate and MRI-based activity in patients switched from 30 mcg to 44 mcg of IFNβ-1a (12). The INCOMIN study reported, compared to IFNβ-1a (30 mcg once a week as an intramuscular injection), a higher rate of relapse-free patients and a smaller number of new T2 lesions based on MRI for treatment with IFNβ-1b (250 mcg every other day as a subcutaneous injection) (13). Koch-Henriksen et al., who compared the effect of IFNβ-1a (22 mcg 3 times a week as a subcutaneous injection) and IFNβ-1b (250 mcg every other day as a subcutaneous injection), found no difference in the relapse rate over 24 months or in the time to first relapse (14). The QUASIMS study found no significant difference in the annualized relapse rate (0.51 for IFNβ-1a 30 mcg, 0.52 for IFNβ-1b, 0.53 for IFNβ-1a 22 mcg, and 0.63 for IFNβ-1a 44 mcg) (15). In the REGARD study, there was no significant difference between groups in the time to first relapse treated with GA 20 mg daily and IFNβ-1a 44 mcg 3 times a week (hazard ratio 0.94, 95% CI 0.74–1.21; *p* = 0.64) (16). A slightly lower relapse incidence was found among patients treated with glatiramer acetate or subcutaneous IFNβ-1a relative to intramuscular IFNβ-1a and IFNβ-1b (17).

These findings and our data suggest that the evaluation of drug effects may be complicated by several factors. Our study analyzed MS patients after the first demyelinating event. A limitation of this analysis is omitting the initial MRI activity as an important predictive and prognostic factor. Furthermore, we did not investigate the presence of IFNβ-neutralizing antibodies (or myxovirus resistance protein A) that could also affect the biological activity of IFNβ (18). Compared to other small registry-based observational studies, an advantage of this study is the relatively high proportion of patients (hundreds of patients in the first 4 groups).

In a multicentric study with a large number of patients, we cannot be sure that the sample differences in EDSS at baseline may be due to bias during the treatment selection process. Indeed, the differences between GA and IFNβ-1a 44 mcg in ARR in the follow-up became non-significant after including age and EDSS score at baseline. This could be seen as a limitation to our data interpretation. Nonetheless, in our study, we sought to avoid the well-known biases of observational studies [compared to randomized controlled trials (RCTs)] as much as possible. We tried to avoid selection bias through validation analysis performed by employing multiple regression models adjusted for the confounding factors of age and EDSS score at baseline. An alternative approach to reduce the effect of confounding factors consists of comparing the results with the validation analysis performed in matching groups, the so-called propensity score matching method, used, e.g., by Kalincik (19). This matching approach would not be suitable for our study given that only 5 groups were compared. Furthermore, using the teriflunomide group as a reference, for example, the samples of the other groups would not be representative. If another of the 4 remaining DMDs were used as a reference, the number of patients in the other groups would be substantially reduced (particularly in the teriflunomide group), and the statistical power of inductive assessments would substantially decrease. Elimination of the teriflunomide group would be a possible solution; however, this would result in losing important information and would consequently be detrimental to this analysis.

Bias was further reduced in our study by including patients who satisfied the relatively strict inclusion criteria. The purpose of these strict criteria was to ensure that the groups were as homogeneous as possible in terms of covariates. The patients were selected from the ReMuS registry, which contains national, good-quality data prospectively controlled since 2013. The registry contains information on approximately 70% of patients in the Czech Republic. At the same time, the registry uses uniform methodologies of input and data quality control from all MS centers, which is an advantage compared to large pooled studies (e.g., from MSBase), where patient data are provided by centers in various countries using non-uniform methodologies. Thus, there is a smaller risk of information bias from the ReMuS data. The reimbursement criteria for DMD therapies differ in various countries; unfortunately, this has an impact on the correctness and inconsistencies in the EDSS values and relapse incidences reported to the registries. Insufficiently reported CDP and ARR values may thus result when compared to RCTs (20). To obtain statistically powerful inductive assessments, an appropriate follow-up period is recommended, such that the studied event occurs in at least 50% of cases. In our case, relapse occurred “only” in 36.5% of patients and CDP in 5.3% of patients during the chosen follow-up period. A longer follow-up period would be advisable, particularly for the time to CDP, to obtain more statistically robust results. We opted for a shorter follow-up period – 2 years – to ensure that the effect of DMDs on the studied clinical parameters of disability would be clearer and more unquestionable. Nonetheless, we are aware that this relatively short follow-up period may have prevented the appearance of additional EDSS worsening events.

In addition, given the large number of patients, at this stage, we could not include more sophisticated clinical and functional data or MRI data. For these reasons, our analysis should be viewed as prospective, and the inductive conclusions should be validated by a similar observational study with a longer follow-up period and additional clinical and MRI data, taking into account the potential decrease in the net effect of the first DMD with time.

We also found that the group of patients on teriflunomide was significantly different in terms of age and sex distribution. This group included more males, and the patient age was higher. The reason for this sex disparity is probably related to the embryotoxic and teratogenic potential of teriflunomide. Thus, this drug was not as commonly prescribed to females of reproductive age (the main patient cohort with the first detection of MS). However, according to a recent study by Vukusic et al. analyzing data from female patients exposed to teriflunomide during conception or pregnancy, no significant differences were found when comparing these data to the healthy population data (21).

Additionally, the reasons leading to the termination of therapy were analyzed. As confirmed by statistical testing, compared to the other groups, the proportions of reasons to terminate DMD therapy differed for GA. GA therapy was most commonly terminated for intolerance because of post-injection complications, while in the other DMDs, the most common reason was a lack of effectiveness of the therapy.

GA was one of the first drugs to be approved for MS treatment, and its tolerability has been well-established by numerous clinical studies (22). Compared to other drugs, the finding that in our sample, there is a higher percentage of patients who terminate GA therapy for post-injection complications is probably due to the mechanism of action of this medication. Although this mechanism is not yet fully understood, it is assumed that it includes the activation of various pathways of both the innate and adaptive immune systems (23). It is known that in some cases, GA can induce a local injection-site reaction, including tenderness, pruritus, erythema, or induration. In most cases, these phenomena begin immediately after GA injection and resolve spontaneously within a few minutes without any sequelae (22). However, some of these post-injection reactions may remain permanent and lead to discontinuation of therapy (22, 23). It is difficult with current knowledge to determine which patients are at greater risk of interruption. The usefulness of this sample study lies in the observation that in a very large group of patients, GA can be stopped because of these types of adverse reactions more frequently than other drugs.

The lack of therapeutic effects with other DMDs is also difficult to explain. Certainly, the complexity of immunological phenomena during the development of the disease and the modifications induced by the treatments play a fundamental role in the individual response to treatment (24). At present, given the availability of newer oral drugs (25), many clinicians are used to switching therapy if there is no evidence of disease modifying action (23).

In any case, these data indicate that differences in side effects among MS drugs may exist and lead to termination of a given therapy. As a consequence, in clinical practice, it is advised to take into account all potential risks of long-term therapy when choosing a drug (20, 26, 27). The patient should also be involved in the decision-making process, and the patient's preference regarding the administration mode and dosing frequency should be considered. Specific situations that may prevent continued therapy need to be monitored during the treatment: local cutaneous reactions, difficulty breathing and chest pain for GA, flu-like syndromes, cutaneous reactions, depression and hepatopathy for IFNβ, and hepatopathy, diarrhea, nausea and alopecia for teriflunomide.

## Conclusions

Based on the data obtained from the ReMuS registry over a period of 2 years of follow-up, no significant differences were observed for the following clinical parameters: time to first relapse, EDSS score changes, and the rates and time of CDP development. The only significant difference observed was a higher percentage of relapse-free patients in subjects treated with GA than in those treated with IFNβ-1a 44 mcg, but these data were not confirmed by the validation analysis. On the other hand, GA treatment was more often administered than the other drugs to interrupt therapy because of post-injection complications. Thus, although these drugs may be seen as comparable, the choice of therapy should also account for factors such as duration of the therapy, associated risk factors, individual clinical history and current status.

## Data Availability Statement

Raw data is available only with approval from the data owner register ReMuS. Researchers can request for data from register ReMuS directly by applying on: https://www.nfimpuls.cz/index.php/en/czech-ms-registry/about-the-registry.

## Ethics Statement

The studies involving human participants were reviewed and approved by All Ethical Committees for all 15 MS centers in the Czech Republic. The patients/participants provided their written informed consent to participate in this study.

## Author Contributions

ZP, MVal, and LS conceived and designed the data plan. LS and ZP conducted the analyses. ZP, FA, and MVal led the writing of the manuscript. ZP had final responsibility for the decision to submit for publication. All authors provided critical feedback and helped shape the research, analysis, manuscript, had full access to the study data for interpretation and drafting of the report, and contributed to the interpretation of the findings.

## Conflict of Interest

The authors declare that the research was conducted in the absence of any commercial or financial relationships that could be construed as a potential conflict of interest.

## Supplementary Material

Supplementary Figure 1 **(A,B)** Interaction plot for ARR with IFNβ-1a 44 mcg a GA treatment as a factor and confounding covariates EDSS and age. Interaction plots show the relationship between EDSS (plotted on *x* axis), ARR (*y* axis), and age (color of dots) separately for treatment groups IFNβ-1a 44 mcg (left panel) and GA (right panel) and the fitted regression lines (OLS) for 3 age groups. There is one outlier (ARR >3) in the GA group circled in **(A)**, which influences the estimates of fitted lines.

Supplementary Table 1 The Ordinary Least Squares (OLS) models to analyze interaction between IFNβ-1a 44 mcg and GA groups for age and treatment effect (EDSS at baseline for ARR).
