# Supplementary material for: Comparison of Therapies in MS Patients After the First Demyelinating Event in Real Clinical Practice in the Czech Republic: Data From the National Registry ReMuS
Source: Front Neurol. 2021 Jan 12;11:593527. doi: 10.3389/fneur.2020.593527 (PMC7835499; doi:10.3389/fneur.2020.593527)
Supplement: Supplementary Table 1 — The Ordinary Least Squares (OLS) models to analyze interaction between IFNβ-1a 44 mcg and GA groups for age and treatment effect (EDSS at baseline for ARR). [file Table_1.docx]

**Supplementary Table 1.** The Ordinary Least Squares (OLS) models to analyze interaction between IFNβ-1a 44 mcg and GA groups for age and treatment effect (EDSS at baseline for ARR)

| **Coefficient** | **OLS1 (all observations)** | | | **OLS2 (ARR < 3)** | | |
| --- | --- | --- | --- | --- | --- | --- |
|  | *Estimate* | *SE* | *P value* | *Estimate* | *SE* | *P value* |
| Intercept | 0.4411 | 0.1722 | 0.0106 | 0.4410 | 0.1696 | 0.0094 |
| EDSS | 0.1171 | 0.0752 | 0.1200 | 0.1171 | 0.0741 | 0.1143 |
| **age** | -0.0103 | 0.0050 | **0.0422** | -0.0103 | 0.0050 | **0.0391** |
| GA | -0.4949 | 0.2349 | **0.0355** | -0.2812 | 0.2345 | 0.2306 |
| EDSS:age | -0.0001 | 0.0021 | 0.9586 | -0.0001 | 0.0020 | 0.9580 |
| EDSS:GA | 0.1418 | 0.1123 | 0.2075 | -0.0091 | 0.1139 | 0.9359 |
| age:GA | 0.0120 | 0.0068 | 0.0830 | 0.0070 | 0.0068 | 0.3064 |
| EDSS:age:GA | -0.0033 | 0.0031 | 0.2774 | 0.0001 | 0.0031 | 0.9686 |

Legend: OLS1 model was fitted for all observations in IFNβ-1a 44 mcg and GA groups. OLS2 model was estimated after removing one outlier observation with ARR > 3 influencing the analysis. OLS analyses were performed using IFNβ-1a 44 mcg as the reference group. Interaction terms between explanatory variables are denoted by colon SE: standard error.
